# Supplementary material for: Are carbon emissions trading and green financial instruments synergistic? -Comprehensive quantitative research based on content analysis
Source: PLoS One. 2024 Mar 7;19(3):e0298601. doi: 10.1371/journal.pone.0298601 (PMC10919601; doi:10.1371/journal.pone.0298601)
Supplement: S1 Data — (ZIP) [file pone.0298601.s001.zip › Data/地级市Z_F工作报告.zip!/╣≤╓▌/░▓╦│╩╨/└┤╫╘╣1⁄2╓┌║┼í╛┬φ┐╦╩2╛▌═°í┐_2011.docx]

各位代表：

现在，我代表市人民政府向大会作报告。根据《中共安顺市委关于制定安顺市国民经济和社会发展第十二个五年规划的建议》，市人民政府制定了《安顺市国民经济和社会发展第十二个五年规划纲要（草案）》，请各位代表连同政府工作报告一并审议，并请市政协委员和其他列席会议的同志提出意见。

一、“十一五”时期国民经济和社会发展回顾

过去五年，在省委、省政府和市委的领导下，在市人大、市政协的监督、支持下，我们坚持以邓小平理论和“三个代表”重要思想为指导，全面贯彻落实科学发展观，紧紧抓住西部大开发和新阶段扶贫开发的机遇，团结和依靠全市各族人民，聚精会神搞建设，一心一意谋发展，克服了全球性金融危机的严重影响，战胜了百年不遇的雪凝冰冻、特大重旱和“6·28”滑坡泥石流等自然灾害，积极应对经济社会发展中的各种困难和挑战，完成了“十一五”规划的主要目标任务，进入了全面建设小康社会新的历史时期。

——过去的五年，是综合实力显著提升，经济持续快速发展的五年。主要经济指标实现翻番。全市地区生产总值由2005年的106.03亿元，增加到2010年的232.92亿元，年均增长12.3%。人均生产总值由2005年的499美元，增加到2010年的1366美元。全社会固定资产投资由2005年的47.11亿元，增加到2010年的111.34亿元，年均增长18.8%。财政总收入由2005年的14.7亿元，增加到2010年的48.6亿元，年均增长27%。地方一般预算收入由2005年的7.44亿元，增加到2010年的19.84亿元，年均增长21.7%。社会消费品零售总额由2005年的28.45亿元，增加到2010年的70.08亿元，年均增长19.8%。“十一五”期末，金融机构存贷款余额分别为362.19亿元、229.65亿元，是“十五”期末的2.6倍和1.9倍。

——过去的五年，是基础设施建设明显加快，发展条件不断改善的五年。我们以交通、水利为重点，加强基础设施建设，着力夯实发展的基础和改善发展的条件。综合交通运输条件得到很大改善。黄织铁路建成通车，长昆铁路客运专线顺利开工，境内3个站点的规划建设同步推进，铁路通车里程192.2公里，在建里程173.3公里。坝陵河大桥顺利开通，沪昆高速公路安顺段建成通车，普定至安顺及安顺西绕城高速公路、惠兴高速公路建设稳步推进，高速公路通车里程136.1公里，在建里程152公里。建成通乡油路495.2公里、通村公路4616.6公里，实现所有乡镇通油路（水泥路），所有行政村通公路。公路通达里程9150公里，公路密度达到每百平方公里96.9公里，高于全省平均水平16公里。坝草码头改扩建竣工。黄果树机场达到国家4C级标准。全市初步形成公路、铁路、水运、航空“四位一体”的立体交通网络格局，成为全省交通最为便捷的地区之一。以解决工程性缺水为重点的水利设施建设取得新成绩。黔中水利枢纽、滋黔一期、安西灌区节水续建配套、引千入虹等重点水利工程建设进展顺利，治理病险水库50座，建成一批“三小”水利工程，新增有效灌溉面积29.4万亩，解决了70.1万人的饮水安全。电力、通信等基础设施建设不断加强，城镇居民每百户手机、电脑拥有量从2005年的107部和17台，增加到2010年的171部和47.5台，所有行政村实现通电、通广播电视和通移动电话。

——过去的五年，是三次产业协调发展，经济增长质量逐步提高的五年。我们着力把资源优势转化为经济优势，不断发展壮大三次产业，三次产业结构比重由2005年的22.7：36.9：40.4调整为2010年的17.4：38.0：44.6。农业农村经济稳步增长。粮食产量稳定在80万吨左右。农业结构调整成效显著，经济作物种植规模进一步扩大，产量和效益不断提高。畜牧业产值占农林牧渔业总产值的45.1%，年均提高1.4个百分点，在全省保持领先地位。农业产业化步伐加快，成功引进雨润集团、柳江集团等大型龙头企业，全市现有国家级龙头企业1家，省级龙头企业20家，市级龙头企业87家，有力地带动了农村经济发展。扶贫开发成效显著，减少农村贫困人口13万人。新农村建设取得明显成效，完成134个新农村试点示范村建设。

工业经济规模效益显著提升。能源、原材料等优势产业进一步壮大，光照电站、董箐电站建成发电，马马崖电站开工建设，原煤产量逐年增加，超宇水泥、黄果树铝业、宏盛化工等企业发展迅速，全市发电量达到87.1亿千瓦时，水泥产量达到223.9万吨。以航空、汽车为重点的装备制造业稳步发展，民用航空产业国家高技术产业基地落户安顺，天马轴承、青年莲花等企业顺利投产。以食品、药品为重点的特色轻工业发展加快，百灵集团成功上市、安酒公司重组恢复生产。全市纳入地方统计年主营业务收入500万元以上的工业企业达215家，较“十五”末增加97家，总产值达155.1亿元，是“十五”末的2.46倍。工业园区建设初见成效，安顺经济技术开发区、民用航空产业国家高技术产业基地、西秀工业园区、黎阳高新区夏云工业园区、普定循环经济工业基地等园区配套基础设施建设力度加大，入驻企业达347户，2010年实现工业总产值97.5亿元。

以旅游业为龙头的第三产业加快发展。景区景点保护开发力度加大，黄果树、龙宫成功申报为国家首批5A级旅游景区，夜郎洞成功申报为4A级旅游景区。旅游基础设施建设成效显著，一小时旅游交通圈基本形成，星级酒店建设步伐加快，三星级以上酒店发展到12家，在建五星级酒店达到4家。城市知名度和美誉度得到提升，黄果树瀑布节成为国内极具影响力的旅游节庆活动，安顺荣膺“中国优秀旅游城市”、“中国十大特色休闲城市”。2010年，全市接待游客1324.8万人次，实现旅游收入112.8亿元，分别是2005年的2.7倍和10倍。在旅游业的带动下，金融保险、交通物流、邮电通信、信息服务、酒店餐饮等第三产业快速发展。2010年第三产业增加值突破100亿元，达到103.9亿元。

——过去的五年，是城镇化进程加快推进，城乡面貌大幅改变的五年。我们围绕建设百万人口生态旅游城市的目标，加快推进城镇化进程，全市城镇化率由2005年的28.5%提高到2010年35%，年均提高1.3个百分点。城市规划体系进一步完善，新修编的城市总体规划和土地利用总体规划通过省政府批准。城市路网骨架基本成型，新建改造道路81.6公里。中心城市建设步伐加快，建成区面积由“十五”末的26.7平方公里扩展到32.5平方公里。主城区旧城改造不断推进，启动了中华西路片区、北山片区、安普路片区棚户区改造，治理小街小巷33条。市政公用设施建设力度加大，供水管网改造、电网入地、天然气普及工作稳步推进，金钟、若飞、文庙、南马、百灵广场和虹山湖休憩区建成开放，电影城、体育场投入使用，实施绿化改造工程18项，城区绿化覆盖率达到31.8%。各县城加快发展，城市功能不断完善，作为县域中心城市的辐射带动能力明显增强。小城镇规划建设稳步推进，一批富有地方特色，具有一定产业基础的小城镇呈现出蓬勃发展的态势。全国文明城市、国家卫生城市、国家环保模范城市创建工作深入开展，城乡文明程度显著提高。

——过去的五年，是环境保护力度不断加强，生态环境明显改善的五年。我们切实加大环境保护力度，强力推进节能减排，生态环境不断改善。市和各县区污水处理厂建成并投入试运行，日处理生活污水能力达8.3万吨。各县城垃圾填埋场建设扎实推进。退耕还林和天然林保护工程成效显著，完成营造林149.6万亩，森林覆盖率由2005年的33.1%提高到2010年的38%。水土流失治理、石漠化治理工作有效开展，治理小流域43条，治理水土流失面积413平方公里，综合治理石漠化面积161.1平方公里。城市环境空气质量优良率保持100%，集中式饮用水源全面达标。

——过去的五年，是改革开放持续深入，发展动力显著增强的五年。我们认真总结安顺试验区成立20周年的实践经验，积极探索“贵州加快发展的经济特区”的发展途径，不断深化安顺多种经济成分共生繁荣试验区改革。市、县区政府机构改革顺利完成，政府职能转变加快。市与县区分税制改革成功实施。城市商业银行挂牌成立。市属国有企业改制工作基本完成。农村土地承包经营权流转稳步开展，集体林权制度主体改革顺利推进。文化、医疗卫生体制改革进一步深化。非公有制经济不断发展壮大，全市非公有制经济占地区生产总值的比重达52.5%。全面推行政务公开，简化行政审批，强化效能监察，深化民主评议行风，发展环境不断改善。招商引资工作力度加大，累计引进资金208.8亿元。开展多种形式的对外交流，与“珠三角”、“长三角”地区、对口帮扶城市和友好合作城市的交流与协作进一步加强，“九顺合作”、“农安合作”取得明显成效。

——过去的五年，是民生工作力度不断加大，人民生活水平日益提高的五年。我们坚持把保障改善民生作为一切工作的出发点和落脚点，认真解决好事关人民群众切身利益的问题，努力实现发展成果由人民共享。城镇居民人均可支配收入由2005年的7337元增加到2010年的14504元，年均增长14.6%；农民人均纯收入由2005年的1828元增加到2010年的3526元，年均增长14%。坚持实施积极的就业政策，全面促进就业和再就业，累计新增城镇就业7.53万人，城镇登记失业率控制在4.5%以内。新型农村合作医疗实现全覆盖，参合率达到97.5%。城乡社会保障体系和社会救助体系进一步完善，凡是符合条件的对象全部纳入城乡低保覆盖范围，实现应保尽保。新型农村社会养老保险试点工作稳步推进。城镇居民基本医疗保险制度全面实施，医疗救助和临时救助制度实现全覆盖。建成廉租房20.71万平方米、经济适用房43.4万平方米，解决了10806户城镇低收入家庭住房困难问题。完成农村危房改造3.79万户。

——过去的五年，是社会事业全面发展，经济社会建设协调推进的五年。我们在大力发展经济的同时，切实加强社会建设和管理，努力构建和谐社会。教育事业快速发展，新建改扩建学校503所，建成校舍面积68万平方米；城乡免费义务教育全面实施，“普九”人口覆盖率达100%；学前教育、高中阶段教育健康发展，职业教育规模不断扩大，高等教育改革顺利推进，人民群众受教育程度不断提高。科技工作成果显著，在全省率先进入省级科技进步先进市行列。知识产权事业健康发展，成功申报为国家知识产权试点城市。文化事业欣欣向荣，文化体制改革取得明显成效。医疗卫生服务能力得到提高，城市社区和县乡村三级卫生服务体系进一步完善。医药卫生体制改革进展良好，国家基本药物制度得到全面实施。人口和计生工作新机制建设取得成效，人口自然增长率由2005年的7.72‰下降到2010年的6.15‰。人事人才工作进一步加强。全民健身运动广泛开展。“平安安顺”建设扎实推进，信访和安全生产监管工作不断加强，保持了良好的安全稳定形势。民主法制和精神文明建设深入推进。国防建设与经济建设协调发展。防灾减灾工作得到高度重视。消防、食品药品卫生、重大疾病防控等公共安全工作力度加大。双拥优抚、老干、档案、民族宗教、史志、外事、侨务、对台、人防、气象等工作取得新成效。

——过去的五年，是政府职能转变加快，行政效率大幅提升的五年。我们自觉接受人大的法律监督和工作监督，自觉接受政协的民主监督，认真办理人大代表建议598件，政协委员提案913件，做到了件件有答复，事事有回音。认真听取各民主党派、工商联和无党派人士提出的意见和建议，不断改善政府工作。积极支持工会、共青团、妇联、科协、残联、红十字会等人民团体依法按章履行职责。全面贯彻《政府信息公开条例》，切实推进行政权力公开透明运行。广泛开展机关效能建设，建立了政务公开制、首问责任制、服务承诺制，加大了行政执法责任制和问责制落实力度。电子政务建设加快推进。应急管理工作进一步加强，预防处置突发公共事件的能力得到提高。严格落实《廉政准则》和党风廉政建设责任制，加大反腐倡廉力度，加强对重点环节和重点部位权力行使的制约和监督，切实做到为民、务实、清廉。

刚刚过去的2010年，我们全面完成了市二届人大五次会议确定的各项任务。地区生产总值增长12.1%；全社会固定资产投资增长32.2%；财政总收入增长47.8%，地方一般预算收入增长22.6%；社会消费品零售总额增长19%；城镇居民人均可支配收入和农民人均纯收入分别增长8.8%和13.4%。

通过五年来的实践，我们更加深刻地体会到，做好安顺的各项工作，必须坚持以科学发展观统领全局，不断深化对安顺发展规律的认识，紧密结合实际，找准发展定位，理清发展思路，明确发展方向，集中力量解决经济社会发展中的大事难题，创造性地开展工作。五年来的实践，积累的经验弥足珍贵，一是必须坚持大力发展工业经济，切实提高资源就地转化率，努力延长产业链，做大做强产业。二是必须坚持把加快城镇化进程作为解决“三农”问题的有效途径，充分发挥城市辐射带动能力，统筹城乡协调发展。三是必须坚持保障和改善民生，让改革发展成果惠及全体人民，充分调动人民群众建设安顺的积极性、主动性和创造性。四是必须坚持改革开放，把制约发展的难点作为改革的重点，加快重要领域和关键环节改革步伐，全面提高开放水平，构建充满活力、富有效率、更加开放、有利于科学发展的体制机制。五是必须坚持高举发展、团结、奋斗的旗帜，和衷共济，凝心聚力，目标同向、行动同步，同心同力同拍共谋发展。

各位代表！“十一五”期间我市经济社会发展取得的成绩，为“十二五”又好又快、更好更快发展奠定了坚实基础。这些成绩的取得，是省委、省政府正确领导，省直各部门大力支持的结果，是全市各级各部门全面落实科学发展观的结果，是全市各族干部群众抢抓机遇、艰苦奋斗的结果。在此，我代表市人民政府，向为安顺经济社会发展作出艰苦努力和付出辛勤劳动的全市广大工人、农民、知识分子、干部，向各民主党派、工商联、无党派人士和各人民团体，向驻安人民解放军、武警官兵、公安政法干警、民兵和预备役人员，向所有关心和支持我市改革开放和现代化建设的各界人士，表示衷心的感谢并致以崇高的敬意！

在肯定成绩的同时，我们也清醒地认识到，我市发展中还存在一些困难和问题，主要是：发展速度慢，经济总量不大；工业经济“短板”，拉动发展的能力不强；城镇化进程步伐缓慢，辐射带动能力不足；城乡低收入群体生活仍然比较困难，人民生活水平不高；政府职能转变还不到位，政府行政效能和服务管理水平有待进一步提高。这些问题和困难，都需要我们在今后的工作中采取有力措施，认真加以解决。

二、“十二五”时期国民经济和社会发展的主要目标和任务

“十二五”时期，是我市全面建设小康社会的关键期，是加快推进工业化和城镇化的攻坚期，也是我市抓机遇、提速度、上台阶的黄金期，我们努力奋斗的预期目标是：地区生产总值年均增长16％以上；财政总收入和一般预算收入年均增长15％以上；全社会固定资产投资年均增长30％以上；社会消费品零售总额年均增长18％以上；城镇居民人均可支配收入年均实际增长10％以上，农民人均纯收入年均实际增长12％以上；非公有制经济占生产总值的比重达到60%；城镇化率达到40％以上；森林覆盖率达到45％；人口自然增长率控制在6‰以内。

——经济实力显著增强。到2015年，全市地区生产总值确保在2010年的基础上翻一番以上，力争突破500亿元，人均生产总值接近全省平均水平，主要经济指标在西部地级市中的排名实现赶超进位。

——基础设施更加完善。实现县县通高速，县县通铁路，南下航道通江连海，机场航运广达四方，“四位一体”的现代综合交通运输网络体系全面形成。城乡供水安全保障体系逐步完善，工程性缺水问题得到基本解决。“数字安顺”建设成效明显，现代信息网络体系趋于完善。

——三次产业发展壮大。工业增加值年均增长23%以上，到“十二五”末，工业占全市生产总值的比重达40%以上，工业成为全市经济提速发展的火车头。旅游业实现转型升级，旅游资源优势充分转化为经济优势，旅游总收入达到310亿元以上。物流、金融、保险、会展等第三产业快速发展。农业产业结构更加优化，农业效益稳步增加，农林牧渔业总产值达到100亿元以上。

——城乡发展协调推进。中心城市建成区面积达到58平方公里以上，人口规模达50万人以上，城市规模明显扩大，城市风貌更加突出，县城和小城镇加快发展，辐射带动能力显著增强，基本形成一个核心、一个组群、两个支点、两条轴线的城镇体系。农业发展方式加快转变，农村基础设施和公共服务明显改善，农民增收渠道进一步拓宽，社会主义新农村建设取得明显成效，城乡统筹发展开创新局面。

——生态环境持续改善。石漠化治理和生态建设取得明显成效，森林覆盖率年均增长一个百分点以上。资源综合利用水平显著提高，单位生产总值能耗和物耗水平明显下降，主要污染物排放总量减少，生态环境质量持续提高。全民生态环保和节能降耗意识进一步增强，资源节约型、环境友好型社会建设步伐加快，天更蓝、水更清、山更绿，人与自然更加和谐的理想人居环境逐步形成。

——社会事业全面加强。覆盖城乡居民的基本公共服务体系逐步完善，全民受教育程度稳步提升，科学文化素质和健康素质不断提高，文化事业和文化产业加快发展，低生育水平继续保持稳定，民主法制建设得到加强，人民群众合法权益得到切实保障，社会更加和谐稳定。

——居民收入明显提高。实现城乡居民收入增长和经济发展同步，城镇低收入者收入明显增加，农村贫困人口显著减少，城镇居民人均可支配收入达到20000元以上，农民人均纯收入达到6000元以上，人民群众生活更加宽裕，家庭财产普遍增加，生活质量明显提高。

——改革开放不断深入。经济社会发展重要领域和关键环节的改革取得明显进展。政府职能加快转变，政府公信力、执行力和服务水平进一步提高。对外开放不断拓展，形成全方位、宽领域、多层次的对外开放格局，改革、开放、合作更具发展活力、动力。

各位代表！未来五年的目标任务已经确定，只要我们坚定信心，把思想统一到发展上，把心思集中到发展上，把力量凝聚到发展上，抢抓机遇，乘势而为，宏伟的蓝图就一定能变成美好的现实！

（一）实施工业强市战略，增强提速发展的牵引力。坚持把工业作为推进全市经济社会发展的突破口，把园区建设作为工业经济发展的突破口，迅速膨胀工业经济总量，新增规模以上工业企业200家以上，工业总产值突破600亿元，拉动全市经济加快发展。规划建设以装备制造、高新技术、民族制药、特色食品等为主导产业的17个产业园区，形成以线串点、以点带面，县县有园区、园园有特色的产业园区建设格局。加快园区基础设施建设步伐，“十二五”期间，省重点产业园区基础设施建设尽快达到“七通一平”，市级特色产业园区达到“五通一平”。引导企业入驻园区，培育一批规模较大、带动性强的企业集团，形成产业集聚，重点建设产值200亿元以上的民用航空高技术产业基地，产值100亿元以上的西秀工业园区。把装备制造业、能源产业、原材料产业打造成百亿产业，把民族制药、白酒、特色食品等特色轻工业打造成重要支柱产业，加快战略性新兴产业发展，努力将我市建设成为全省重要的装备制造业基地、能源原材料基地和特色轻工业基地。抢抓国家逐步放宽低空空域管制的机遇，大力发展民用航空产业，提升通用飞机、民用无人机等民用航空系列产品的生产能力，形成包括航空制造、通用航空营运、飞行培训、通用飞机维修保障服务的航空产业链，建成面向全国服务的无人机试训基地。

（二）实施城镇化带动战略，增强引领发展的辐射力。围绕建设百万人口生态旅游城市的目标，加快推进城镇化进程，确保城镇化率每年提高一个百分点以上。以规划引领发展，加强规划编制工作，完善县、乡、村各级规划，实现规划全覆盖。做大做美中心城市，坚持新区开发和旧城改造“双轮驱动”，加快中心城市建设改造步伐，以城市快速干道路网建设带动城市规模扩大，力争“十二五”期末，完成城中村改造，初步形成黄果树大街一线和塔山片区、新火车站片区、北部新区、西秀新区、航空城五大组团的框架格局，城市风貌更加彰显山水园林城市特色。加快县城和小城镇发展，推进平坝、普定、镇宁加快与中心城市一体化发展，把关岭、紫云建设成为区域性中心城市，把黄果树新城建设成为黄果树旅游圈的区域旅游集散中心，着力培育一批基础设施条件好、生态环境优良、具有一定产业基础的旅游、工贸、商业服务型的特色小城镇。加快平坝马场一带的产业发展和城市基础设施建设，形成有产业支撑，人居环境良好，公共服务设施配套的城市综合体，成为黔中经济区新兴城市和新的经济增长极。提升城镇综合承载能力，增强城镇公共服务功能，加强教育、卫生、环保、文化、体育、福利、市政等公共服务设施建设，推动社会服务资源向基层和社区转移。创新城镇管理体制和工作机制，积极运用信息化、现代化手段提高城市管理水平。推进户籍制度及其相关配套改革，加快把符合落户条件的农业人口转变为城镇居民。推进一城两区设置和平坝、普定撤县建区工作，加快“镇改办”、“村改居”工作。

（三）加快发展以旅游业为龙头的第三产业，增强优化发展的竞争力。充分发挥我市丰富的旅游资源优势，全力打造黄果树国家公园和国际旅游目的地。加大旅游产品开发力度，整合全市旅游资源，建设黄果树旅游环线、大屯堡旅游区和贯城河沿线城市历史文化、休闲娱乐旅游带，大力开发休闲度假、文化科考、康体养生、红色经典等旅游产品，推动旅游从观光游向休闲度假深度游转型升级。加快生态体育公园建设。大力发展宾馆酒店业，加快高星级宾馆酒店建设，五星级酒店达到10个以上，各县区都有四星级以上酒店，旅游住宿设施床位总数达到7万张。到2015年，接待国内外游客2200万人次以上。大力发展现代物流业，规划建设一批物流园区和专业物流市场，把安顺建成服务黔中经济区、面向泛珠三角和东盟自由贸易区的区域性现代物流中心城市。积极发展金融保险、会展经济、房地产、商贸餐饮、文化娱乐、健身养生、信息咨询、科技服务、社区服务等现代服务业。

（四）加快农业现代化进程，增强协调发展的推动力。始终高度重视“三农”工作，积极发展现代农业，力争农林牧渔业总产值年均增长12%以上。保障和稳定粮食生产，加快农业产业结构调整，扩大特色经济作物种植规模，大力发展现代生态畜牧业，努力发展特色休闲观光农业，积极推进农产品加工业发展，不断提高农业综合效益。大力实施水源工程、灌区工程、“五小”工程，进一步提高农村人均有效灌溉面积，加快建成人均半亩基本口粮田。实施农村公路提级延伸连网工程，大力推广沼气等能源利用，完善农村电网建设。加大扶贫开发力度，不断降低农村贫困面，到2015年农村贫困人口减少18万人以上。加快推进社会主义新农村建设，增加农村社会事业投入，大力实施“六到农家”工程，建设农民幸福生活的美好家园，推动城乡统筹协调发展。

（五）加强基础设施建设，增强跨越发展的支撑力。继续把以交通和水利为重点的基础设施建设作为事关全局的重大战略任务。加快铁路、高速公路建设，实施县乡公路提等改造工程，全市公路油路比率提高到50%以上，建设通村油路3000公里，实现70%的行政村通油路。推进坝草码头和红水河黄金水道建设，开辟南下两广通行500吨级船舶的四级航道。积极争取开通航线，提高黄果树机场的使用效益。完善集公路、铁路、水路、航空“四位一体”的现代综合交通运输体系。大力加强水利基础设施建设，力争今后10年全社会水利年平均投入比2010年高出一倍，推进黔中水利枢纽工程建设，新建黄家湾水利枢纽等16座水库，治理病险水库76座,实施农村饮水安全工程，解决42.5万人的饮水安全，全市城乡饮水安全问题和工程性缺水问题得到基本解决。完善信息基础设施，加快“数字安顺”建设，推进电信网、互联网、广播电视网“三网融合”。

（六）加强生态建设和环境保护，增强可持续发展的承载力。牢固树立绿色、环保、低碳发展理念，切实加强节能减排、生态建设和环境保护，加快构建资源节约、环境友好的生产方式和消费模式，实现安全发展、绿色发展和可持续发展。实施重点节能工程，提高土地资源利用水平，加强水资源保护利用和矿产资源开发管理，大力发展循环经济，提高资源利用率。加强林业生态建设，大力实施生态保护重点工程，扎实推进石漠化综合治理，努力构建功能完备的“两江”生态体系。切实加强环境保护和污染治理，加强大气污染防治和固体物污染治理，努力改善水环境质量，到2015年，城镇生活垃圾无害化处理率达到85%以上，生活污水集中处理率达到80%以上。

（七）深化试验区改革开放，增强率先发展的内生力。用好用足“贵州加快发展的经济特区”的政策，进一步消除制约经济社会发展的深层次体制机制障碍。继续深化行政管理体制、投融资体制、财税体制、社会事业体制改革和农村综合配套改革、林权制度改革、要素市场改革等重点领域改革。积极发展村镇银行和小额贷款公司，建设股权投资和产权交易市场，助推大企业上市融资。大力发展非公有制经济。全方位扩大对内对外开放，加强与珠三角、长三角、京津唐、成渝地区的交流与合作，积极参与中国—东盟自由贸易区经贸合作，加强与对口帮扶城市和友好合作城市的交流与对接，促进合作共赢。扎实做好招商引资项目规划、设计、宣传工作，切实提高招商引资实效，确保“十二五”期间招商引资到位资金达到680亿元以上。坚持下硬功夫改善软环境，进一步优化发展环境。

（八）切实保障和改善民生，增强和谐发展的凝聚力。健全完善覆盖城乡的社会保障体系，逐步实现人人享有基本生活保障的目标。认真落实各项就业政策，多渠道开发就业岗位，努力提高就业率。加快农村危房改造，实施保障性住房建设工程，切实保障广大人民群众的基本住房需求。加快发展各项社会事业，增强公共服务能力，促进社会全面进步。坚持教育优先发展，实施好《安顺市中长期教育改革和发展规划纲要（2010—2020年）》。加强科技创新和科技服务体系建设，提高科技成果转化率。加强人才队伍建设，大力实施各类优秀人才培养工程，广泛引进重点领域、重点产业、重点学科、重点项目急需人才。加快文化产业发展。广泛开展全民健身运动，提高竞技体育水平。健全公共卫生服务体系和医疗服务体系，加强城镇社区卫生服务体系和农村卫生服务网络建设。全面加强人口和计划生育工作，采取有效措施统筹解决人口问题。深入开展“三创”和精神文明创建活动，努力提高全民文明素质。严格落实安全生产责任制，确保安全生产形势稳定。加强防灾减灾体系建设，健全完善公共应急处置机制，提高应对自然灾害和突发事件的能力。高度重视信访工作，及时化解各类矛盾。深入推进“平安安顺”建设，强化社会治安综合治理，依法严厉打击各种犯罪活动，维护社会和谐稳定。

三、2011年经济社会发展的主要目标和任务

各位代表，2011年是实施新一轮西部大开发的第一年，也是“十二五”规划的开局之年，做好今年的各项工作，意义重大。2011年全市经济社会发展的主要预期目标是：地区生产总值增长16%以上；工业增加值增长23%以上；完成全社会固定资产投资258亿元以上；财政总收入和一般预算收入分别增长15%以上；城镇居民人均可支配收入实际增长10%以上，农民人均纯收入实际增长12%以上；社会消费品零售总额增长18%以上；城镇登记失业率控制在4.5%以内；人口自然增长率控制在6.5‰以内；居民消费价格总水平涨幅控制在4%左右；完成省下达的节能减排任务。

围绕实现上述目标，我们将重点抓好以下八个方面的工作。

（一）千方百计抓好项目建设，确保完成258亿元固定资产投资目标任务

加大项目前期工作力度。加强项目库建设，精心谋划项目，优化项目结构，扎实抓好项目前期规划、设计、申报及储备工作，切实解决项目少、项目小、环评难、落地难、推进慢的突出问题，做到开工一批、建设一批、储备一批、谋划一批。今年，市级财政继续安排4000万元作为项目前期工作经费，为项目顺利落地创造条件。

强力推进重大项目建设。重点抓好普定至安顺及安顺西绕城高速公路建设，力争年内建成通车。加快推进安紫高速公路前期工作，力争6月前动工。配合做好我市境内铁路和高速公路的建设工作。抓好长昆铁路客运专线在我市境内三个站点的规划建设。配合做好黔中水利枢纽一期工程建设。完成油菜河水库、鲁嘎水库、板贵引水工程，努力争取坝陵河水库、石珠桥水库、新场河水库工程立项，扎实做好黄家湾水利枢纽工程前期工作。加快推进引千入虹工程。强化项目建设督促检查，严格执行项目建设责任制，建立重大项目进度月通报制度，确保项目建设顺利推进。

进一步拓宽项目融资渠道。充分发挥国资公司、城投公司、交投公司三大融资平台的作用，整合优化资源，建立运作规范的融资体系，增强融资能力。深化政银、银企合作，建立政府引导、银行支持、担保介入、企业参与的融资工作机制，积极开展项目融资，加快融资需求项目库建设，及时将融资项目推介给金融机构。积极通过各种金融工具和衍生工具进行融资，支持企业发行债券、上市融资和上市公司再融资。采取土地一级开发、BOT、TOT、BT等方式拓宽融资渠道。

（二）大力发展工业经济，确保规模以上工业增加值达到70亿元

狠抓工业项目实施。坚持把扩大产业投资作为膨胀工业总量的有效途径，着力抓好100个重点工业项目的实施，完成工业固定资产投资100亿元以上，新增30个规模以上工业企业。加大对中小企业发展的扶持力度，充分发挥政府性资金的引导作用，用好市级财政1000万元中小企业发展资金。

加快园区规划建设。合理规划产业布局，分级分类完成全市17个产业园区控制性

详规和规划编制工作，重点做好黔中新兴产业示范园区、幺铺—黄桶产业园的规划报批工作。围绕实现“七通一平”的目标，加大五大园区的基础设施建设力度。本着“精简、统一、效能”的要求，进一步理顺园区管理体制和管理机构。筹措1亿元资金加快民用航空产业国家高技术产业基地基础设施建设。

优先发展装备制造业。依托民用航空产业国家高技术产业基地，加快发展通用飞机、民用无人机制造等民用航空产业项目，积极引进DA42飞机项目，力争年内实现首飞。扶持青年莲花轿车扩大生产规模，力争年内生产轿车5000辆以上，并积极创造条件，力争“十二五”末年产量达到10万辆。大力发展专用车整车制造和专用机械制造。

大力发展能源工业。加快煤炭企业兼并重组步伐，形成10个以内年产150万吨以上煤炭企业集团。确保安顺电厂三期开工建设。加快盘县至安顺大型带式输送机输煤工程前期工作，尽快开工建设。加快推进北盘江、蒙江河流域梯级电站开发进程。

提升发展原材料产业。加快昌兴、港安、润基等水泥厂建设。抓好黄果树铝业公司10万吨铝锭环保节能技改项目和10万吨铝板带泊深加工建设项目，大力发展铝深加工，努力把黄果树铝业培育成百亿级企业。支持红星集团、宏盛化工等一批企业做大做强。调整优化铁合金产业。大力发展新型墙体材料，支持发展新型节能环保建材和绿色装饰材料。

加快发展特色轻工业。立足我市旅游资源、生物资源和民族文化资源，大力发展具有比较优势的特色食品、民族制药和旅游商品。继续推进安酒技改项目，加快平坝、普定、镇宁等地白酒业发展，重塑“安顺白酒”品牌。充分利用百灵集团成功上市的有利条件，加快推进药企整合，形成以民族制药为核心的生物医药产业集群。推进镇宁特色轻工产业园和黄果树旅游商品加工博览园建设，做大镇宁波波糖、蜡染、傩雕等旅游商品特色产业。实施好华森饮用泉水改扩建项目和黄果树金叶公司烟叶薄片项目。

培育高新技术及战略性新兴产业。重点在高端装备制造、生物技术、新材料、新能源、节能环保等领域加快自主创新及产业化发展。抓好百灵制药原材料生产基地项目建设，打造国家先进水平的苗医药研发平台，推动用于重大疾病防治的医药研发和生产。积极推动以汽车动力电池为代表的新能源汽车配套关联产品研发和生产。

（三）强力推进城镇化进程，确保城镇化率提高1.5个百分点以上

抓好规划编制工作。按照新修编的城市总规和土地利用总规，进一步合理划分城市功能区，优化土地利用空间布局。加快中心城市控制性详规编制进度，实现详规全覆盖。完成镇宁、关岭、紫云县城和黄果树、龙宫风景区总体规划修编工作，加快安顺市城市综合交通体系规划、安顺市城市景观风貌规划等专项规划编制工作，实现乡镇、村寨规划的全覆盖。

加快中心城市建设改造步伐。完成市级行政中心和体育场馆规划，并力争年内开工建设。进一步完善城市路网，拉大城市框架。推进二环路、武当路、北山路、安普路等一批城市主次干道建设，确保中华西路6月底前通车。完成城区主街道“白改黑”工程。加快新区开发建设，启动新火车站片区开发，推动北部新区开发。加大旧城改造力度，抓好城市棚户区改造，启动历史文化街区改造，实施小街小巷综合整治，改造汪家山、管元等“城中村”，努力使老城区面貌有一个大的改观。充分发挥城市建设发展基金的杠杆效应，撬动更多的资金投入城市建设。做好土地这篇文章，节约、集约利用土地，发挥安顺土地资源优势，加快城市融资和建设，各县区“土地增减挂钩”实现重大突破，加大土地整治工作力度，为增加城市建设用地开辟新途径。

推进县城和小城镇发展。进一步加强县城城区道路等基础设施建设，完善城区路网，拓展城市空间。突出抓好规划镇和重点中心镇建设，提高辐射带动能力，带动区域经济协调发展。

着力增强城镇综合承载能力。进一步完善市政设施建设，启动中心城市供水二期工程，抓好供水管网改造工程，增强城市供水安全保障能力。全面完成中心城市和县城污水收集管网建设。启动中心城市生活垃圾收运系统工程和市污水处理厂二期项目建设。抓好各县垃圾填埋场建设，确保年内建成并投入运行。在市区建设一批人行天桥和地下人行通道，缓解城区交通压力。加快市人民医院新院建设，推进西秀、平坝、关岭县级医院标准化建设，完成市二中新校区建设工程并投入使用，启动市全民健身中心、市文化中心和头铺客运站建设，完成市殡仪馆建设并投入使用，不断增强城市服务功能。

提升城市管理水平。强化规划的统一性和权威性，严查严处违法建设行为。严格执行闲置土地处置政策，提高城镇土地利用水平和管理水平。深入推进“三创”工作，加大城市管理力度，努力营造适宜居住和创业的城市环境。

培育黔中经济区新兴城市和新的经济增长极。谋划好我市产业发展的空间布局、资源配置，形成与贵阳资源共享、互为市场、产业互补的发展格局。重点加快与贵阳一体化发展的骨干道路建设，开工建设马场到贵阳党武的城市道路。加快平坝马场黔中新兴产业示范园区的规划建设，积极争取建立航空保税物流区和保税加工区，把平坝马场黔中新兴产业示范园区作为高校、科研单位高新技术成果孵化园区和重要的产业承接基地，打造成为黔中经济区的新兴城市和新的经济增长极。

（四）推进旅游产业化进程，确保接待游客1500万人次、实现旅游收入130亿元

进一步整合旅游资源。重点构建好黄果树旅游环线、大屯堡旅游区和贯城河沿线城市历史文化、休闲娱乐旅游带，形成国际知名的旅游区域，增强安顺旅游在海内外的整体竞争力和吸引力。尽快启动黄果树国家公园规划建设和郎弓景区国际旅游度假项目建设。4月底前全面完成黄果树半边街搬迁，提升黄果树核心景区形象。做好坝陵河大桥景区开发建设工作。10月底前全面完成沪昆高速公路安顺境内沿线建筑景观整治工作。

进一步完善旅游要素。精心设计旅游线路，打造2-5日旅游产品，延长游客停留天数；打造美食一条街，塑造“吃在安顺”的品牌；培育一批地域标志性旅游商品，建设特色旅游商品集中展示购物区；打造具有安顺特色的体育旅游品牌赛事活动和文化旅游精品剧目，鼓励发展保健康体休闲产业，丰富游客在安娱乐活动。充分利用良好的生态气候资源优势，开发建设一批避暑养生度假区。加强景区景点连线建设，提高旅游可进入度。加快改造建设虹山大酒店、国际佳缘大酒店、黄果树宾馆、屯堡酒店、燕安酒店、安都大酒店等一批高星级酒店。

进一步理顺旅游管理体制。积极探索旅游资源一体化管理模式，逐步实现景区统一管理。鼓励社会资本参与我市旅游业发展。扶持发展一批大型旅行社。支持黄果树旅游集团公司上市融资。积极与海航集团合作开发安顺旅游资源。加大航班航线培育的政策扶持力度，实现黄果树机场正常通航。紧紧抓住第九届少数民族传统体育运动会在我省举办的机遇，大力开展旅游宣传营销。用市场化的办法举办旅游节庆活动，在上半年举办好以“壮美大瀑布、避暑康体游”为主题的黄果树瀑布节。

大力发展现代服务业。以幺铺—黄桶产业园建设为龙头，依托综合交通和煤炭产业优势，加快推进大物流建设。探索发展冷链物流、医药物流等专业物流，引导物流业建立供应链服务体系。加强物流资源整合和信息化建设，引导企业进行物流外包，发展引进大型现代物流企业和第三方物流，提升物流的专业化和社会化水平。突出发展金融、保险、中介服务等生产性服务业，推进服务业结构转型升级优化。提升发展商贸服务业，逐步形成以两城区为中心，各县城为结点的商贸网络体系。加快建设红星美凯龙、中天汽车城、关岭农产品批发市场等专业市场，培育一批商品集散功能强、产业关联度高、区域辐射面广的大市场。大力发展商贸餐饮、文化娱乐、健身养生、信息服务、会展等现代服务业。

（五）扎实抓好“三农”工作，确保农民人均纯收入实际增长12%以上

加强农业基础设施建设。抓好人均半亩基本口粮田规划建设，大力实施民生水利工程，治理病险水库27座，完成3.4万亩“烟水配套”工程建设，增加有效灌溉面积8.4万亩，新增解决15万农村人口的饮水安全。

不断优化农业产业结构。确保粮食产量达到80万吨以上，蔬菜产量达到100万吨以上。大力发展茶叶、烤烟、中药材等特色种植业。种植蔬菜70万亩，新建茶园10万亩，种植烤烟9.5万亩，新增种植中药材4.5万亩，新增种植水果5万亩。推进与华西希望集团的合作，建设50万头商品猪基地及相关深加工项目。依托雨润、柳江、牛来香等龙头企业，大力发展现代生态畜牧业，扶持建设一批规模养殖场（小区），确保畜牧业产值占农林牧渔业总产值的比重提高1个百分点以上。加快发展特色农产品加工业，推进农业规模化生产、产业化经营。积极推进农业机械化。鼓励发展特色观光休闲农业，增加农民收入，提高农业综合效益。

加大扶贫开发力度。启动新的10年扶贫开发纲要编制工作。实施好扶贫脱贫攻坚工程，完成易地扶贫搬迁2800人，减少贫困人口3.6万人。拓宽贫困地区群众就业渠道，培训转移800人，转产1万人。实施5个乡、80个村整乡（村）推进工作。大力推进村庄整治工作。加快社会主义新农村建设步伐，力争完成160个“四在农家”创建点建设，完成2600户农村沼气池和10个农村清洁工程示范点建设，促进农村村容村貌有大的改观。

（六）加强生态建设和环境保护，确保森林覆盖率达到39%以上

加强环境保护和生态建设。推进水利建设、生态建设、石漠化治理“三位一体”规划实施，完成石漠化治理面积172.9平方公里，治理水土流失面积100平方公里。继续抓好退耕还林、天然林保护等重点林业工程建设，完成营造林面积14万亩，确保森林覆盖率提高1个百分点。规范矿山环境治理保证金缴存、使用和监管，积极组织实施地质灾害和矿山环境恢复治理工程。继续抓好水污染防治。推进虹山水库及娄家坡水库环境综合整治工作。以中心城市和县城集中式饮用水源地为重点，加强环境污染治理，加快城镇污水处理及再生利用设施和城镇生活垃圾无害化处理设施建设。

加大节能减排工作力度。加大淘汰落后产能力度，加快实施重点节能工程。大力发展循环经济，提高资源综合利用水平。继续围绕重点流域、重点区域、重点企业和重点污染物开展环境整治，严格环境执法，确保二氧化硫等重点污染物的排放总量控制在国家规定的范围之内。

（七）突出抓好和谐社会建设，确保全面完成“十大民生工程”

切实保障和改善民生。我们将把民生摆在更加重要的位置，下大力抓好就业、就学、就医、住房、社保等方面的工作，实现发展成果由人民共享，让全市人民过上更加幸福、更有尊严的美好生活。实施更加积极的就业和再就业政策，促进高校毕业生、农民工、零就业家庭等就业，实现城镇新增就业1.8万人，推荐高校毕业生实现就业8000人，转移农村劳动力就业4万人。进一步完善社会保障体系，基本解决企业未参保家属工、临时工养老保障历史遗留问题，实行城镇职工基本医疗保险和生育保险市级统筹，申报一批新型农村养老保险试点县，进一步完善城乡居民最低生活保障制度。加快“一危三棚”改造和保障性住房建设，新开工建设9803套廉租住房、1309套经济适用住房和380套公共租赁房，基本解决我市人均住房建筑面积在15平方米以下的城镇低收入家庭的住房问题，完成农村危房改造21429户。进一步发挥住房公积金制度在住房保障体系中的积极作用。做好地质灾害的评估、预警预报和防范治理工作，一季度完成全市地质灾害隐患点拉网式排查工作，制定处置措施和经费保障方案。做好受地质灾害威胁的农房和学校搬迁工作。

推动社会事业全面发展。坚持教育优先发展，深入推进教育事业改革，巩固提高“两基”成果，启动实施学前教育三年行动计划，扩大中职、普通高中、高等教育招生规模，实施劳动者素质提高工程。积极推进公立医院改革，所有政府举办的基层卫生医疗机构实施基本药物制度，实行药品零差率销售。建立失地农民和水库移民生活保障制度。稳步提高基本医疗保险待遇，将城镇职工基本医疗保险住院报销比例提高到75%以上，城镇居民提高到65%以上。巩固提高新型农村合作医疗制度，人均筹资标准达到200元，确保参合率在95%以上。深入推进人口和计划生育工作综合改革和“两个统筹”，新建改扩建5个乡镇计生服务站、80个村级计生服务室。深化文化体制改革，促进文化产业发展。加快公共文化体育服务体系建设，建成50个农民文化家园、508个农家书屋、24个社区乡镇（村）农民体育健身中心、24个社区乡镇文化站。加强国防和后备力量建设，做好双拥优抚、外事侨务、民族宗教、对台、人防、档案等工作。

狠抓安全生产和维稳工作。狠抓安全生产各项责任制的落实，强化煤矿和非煤矿山、交通运输等重点领域的安全监管，坚决关闭不符合安全生产条件的企业，坚决遏制重特大事故发生，确保实现“双降”。继续深入开展“平安安顺”创建活动，抓好城市报警和监控系统建设，设立监控点1万个、报警点1000个，加强学校安全保卫工作，加大社会治安综合治理力度，切实保障人民群众生命财产安全。积极做好防灾减灾工作。高度重视信访和维稳工作，充分发挥城乡社区自治组织、社会组织和社会工作者队伍的重要作用，推进城乡社区自治和社会自治的良性发展，促进社会和谐稳定。

（八）着力深化改革开放，确保招商引资实际到位资金达到129亿元以上

深化重点领域改革。继续深化安顺试验区改革。深化财税体制改革，完善财税分配关系。深化投融资体制改革，完善国有资产运营监管体系，提高运营效益。深化农村综合配套改革和林权制度改革，完善农村发展的体制机制。大力推进社会事业和公共服务领域改革。加大对非公有制经济的财税、金融等政策支持力度。引导和鼓励民间资本投资城市基础设施建设，参与矿产资源勘探开发，兴办各类规模化的民营医疗机构，进入保障性住房建设等领域。

加大招商引资力度。坚持把招商引资作为扩大开放的重要抓手，以项目促进招商，以招商带动投资，以投资推动发展。落实招商引资责任制，千方百计完成430亿元的招商引资目标任务，确保实际到位资金不低于目标任务的30%。加强招商引资项目库建设，结合我市“十二五”产业发展规划和工业强市、城镇化带动战略的实施，科学规划一批产业链长、产业幅宽、带动性强的大项目充实项目库。建立和完善招商引资项目信息发布制度，建立网上招商常态制度，各县区建立招商小分队，市直部门建立市县联动的招商工作机制，切实提高招商引资工作效率。充分调动我市骨干企业、优势企业的积极性，支持企业以存量引增量。引导龙头企业和核心企业进行产业链、产业群招商。鼓励在安各类商会、协会等民间组织抱团引商。支持外来投资企业以商招商和二次招商。加强与对口帮扶城市和友好合作城市的交流合作，进行对口帮扶式招商。重点面向长三角、珠三角、京津唐等经济发达地区积极引进项目，承接产业转移，做好对接合作，促进更多项目落户安顺。全力配合省里的重点招商计划，做好香港、贵阳区域的招商活动。

不断改善发展环境。以开展“作风建设年、环境建设年、项目建设年”活动为抓手，营造良好的政务环境、法制环境、服务环境、社会环境。完善市县政务服务中心配套建设，进一步为投资者提供更加便捷、高效的服务。全面推行公开办事和公开承诺制度，实行首问责任、限时办结、服务承诺等制度。取消不合理的投资准入前置条件和涉企收费，减轻企业负担。减少行政审批事项，简化审批程序，提高审批效率，建立重点项目绿色通道。推行企业评议管理服务部门制度，实行动态管理。加大问责力度，对违规审批、违规收费、服务态度差、推诿扯皮、吃拿卡要等影响发展环境的行为严肃追究责任。

四、切实加强政府自身建设

各位代表，完成好“十二五”时期我市经济社会发展各项目标任务，关系到安顺的长远发展，关系到安顺人民福祉，需要全市上下树立更坚强的信念，付出更艰辛的努力。我们将在市委的领导下，按照“团结、务实、勤奋、廉洁”的要求，奋发图强、励精图治。

（一）坚持转变政府职能，不断提高科学发展的能力。强化社会管理和公共服务职能，创新公共服务体制机制，提高公共服务质量和水平。完善经济调节和市场监管职能，减少对微观经济活动的干预，努力为各类经济主体创造机会均等、公平竞争的市场环境和社会环境。紧跟时代脉搏和发展步伐，不断研究发展中的新情况、新问题，积极破解前进中的新困难、新矛盾。规范行政权力运行，简政放权，提高政府行政效率、工作水平和服务质量，形成干事创业、增比进位、创先争优的良好局面。大力推进电子政务，实现办公自动化。努力提高政府工作人员素质，打造一支恪尽职守、精干高效的公务员队伍，切实增强责任感和使命感。

（二）坚持推进依法行政，不断提高促进公平正义的能力。坚持依法科学民主决策，加强制度尤其是行政程序制度建设。进一步规范行政执法行为，严格按照法定权限和程序行使权力、履行职责。加强和改进行政复议工作，依法妥善解决行政争议。自觉接受人大及其常委会的法律、工作监督和政协的民主监督，认真办理人大代表建议和政协委员提案。加强监察、审计等专门监督，主动接受新闻舆论和社会公众监督，让人民赋予的权力在阳光下运行。高度重视民主党派、工商联、无党派人士和各人民团体的意见、建议。

（三）坚持转变工作作风，不断提高执行力和落实力。大兴密切联系群众、求真务实、艰苦奋斗之风，努力营造“风气要正、工作要实、干部要干、发展要快”的政治生态环境，真正把心思放在干事业上，把本领用在促发展上，把功夫下在抓落实上，建立固定资产投资、工业增加值、用电量、工商税收、就业人数等重要经济指标月通报制度，全力推动工作落实。结合开展“三个建设年”、“四帮四促”活动和实施“十大民生工程”，切实解决人民群众最关心最直接最现实的利益问题，解决基层在发展中遇到的困难和问题。深化政务公开，依法扩大公开范围和层次，凡是涉及群众切身利益的重大事项都要向社会公开，让群众知情。尊重人民群众主体地位，集中民智，珍惜民力，凝聚民心，充分调动广大群众干事创业的积极性。坚持勤俭办事、厉行节约，反对挥霍公款和铺张浪费，把有限的资金和资源更多用在发展经济、改善民生上。

（四）坚持加强反腐倡廉，不断提高廉洁自律的能力。严格落实党风廉政建设责任制，不断完善教育、制度、监督并重的惩治和预防腐败体系。强化对政府公务人员，尤其是领导干部的理想信念和廉洁自律教育，健全公共资源配置、公共资产交易、公共产品生产等领域的管理制度。建立完善行政审批电子监察系统。继续开展领导干部经济责任审计，努力纠正和查处损害群众利益的不正之风，保持惩治腐败的高压态势，严肃查处违纪违法案件，用实际行动和工作业绩，努力建设团结、务实、勤奋、廉洁的政府，建设有所作为、值得人民群众信赖、让全市人民群众满意的政府！

各位代表！“十二五”的新篇章已经翻开，新的征程赋予了新的使命，新的目标孕育着新的希望。安顺发展已经进入一个新的历史时期，宏伟的事业激励着我们，人民的期待鞭策着我们，美好的前景鼓舞着我们，让我们在市委的坚强领导下，紧紧依靠和团结全市各族人民，坚定信心、扎实工作，开拓进取、奋力拼搏，为开创全市经济社会又好又快、更好更快发展的崭新局面而努力奋斗！
